# QTL mapping of melon fruit quality traits using a high-density GBS-based genetic map

**DOI:** 10.1186/s12870-018-1537-5

**Published:** 2018-12-04

**Authors:** L. Pereira, V. Ruggieri, S. Pérez, K. G. Alexiou, M. Fernández, T. Jahrmann, M. Pujol, J. Garcia-Mas

**Affiliations:** 1 Centre for Research in Agricultural Genomics (CRAG) CSIC-IRTA-UAB-UB, Campus UAB, 08193 Cerdanyola, Barcelona, Spain; 2 IRTA (Institut de Recerca i Tecnologia Agroalimentàries), Campus UAB, 08193 Cerdanyola, Barcelona, Spain; 3 Semillas Fitó S.A., 08348 Cabrera de Mar, Barcelona, Spain

**Keywords:** Quantitative trait loci, Melon, Fruit quality, Fruit morphology, Genotyping-by-sequencing, Genetic map

## Abstract

**Background:**

Melon shows a broad diversity in fruit morphology and quality, which is still underexploited in breeding programs. The knowledge of the genetic basis of fruit quality traits is important for identifying new alleles that may be introduced in elite material by highly efficient molecular breeding tools.

**Results:**

In order to identify QTLs controlling fruit quality, a recombinant inbred line population was developed using two commercial cultivars as parental lines: “Védrantais”, from the *cantalupensis* group, and “Piel de Sapo”, from the *inodorus* group. Both have desirable quality traits for the market, but their fruits differ in traits such as rind and flesh color, sugar content, ripening behavior, size and shape. We used a genotyping-by-sequencing strategy to construct a dense genetic map, which included around five thousand variants distributed in 824 bins. The RIL population was phenotyped for quality and morphology traits, and we mapped 33 stable QTLs involved in sugar and carotenoid content, fruit and seed morphology and major loci controlling external color of immature fruit and mottled rind. The median confidence interval of the QTLs was 942 kb, suggesting that the high density of the genetic map helped in increasing the mapping resolution. Some of these intervals contained less than a hundred annotated genes, and an integrative strategy combining gene expression and resequencing data enabled identification of candidate genes for some of these traits.

**Conclusion:**

Several QTLs controlling fruit quality traits in melon were identified and delimited to narrow genomic intervals, using a RIL population and a GBS-based genetic map.

**Electronic supplementary material:**

The online version of this article (10.1186/s12870-018-1537-5) contains supplementary material, which is available to authorized users.

## Background

Melon (*Cucumis melo* L.) is an important crop worldwide, with a production of more than 31 million tons in 2016 [1]. The main producers are in temperate regions, with China accounting for around 50% of total production. Until the last decade, Africa was considered the origin for melon, but recent phylogenetic studies suggest that the species originated in Asia [2]. Traditionally, two subspecies have been described: *C. melo* ssp. *melo*, which includes most of the commercial varieties in European markets belonging to *cantalupensis* and *inodorus* botanical groups, and *C. melo* ssp. *agrestis*, which contains most of the Asian exotic landraces and accessions [3]. There is high phenotypic and genetic variability between and within melon subspecies for diverse traits, including plant architecture, sex determination, yield and fruit characteristics [3]. Several mapping populations have been used to study this diversity, as F_2_ [4, 5], introgression lines (IL) [6, 7] and recombinant inbred lines (RIL) [8–10]. Generally, the crosses used to develop these mapping populations have been obtained between exotic (*chinensis*, *conomon*, *makuwa* or *flexuosus* groups) and cultivated (*cantalupensis*, *reticulatus* or *inodorus* groups) melon types. However, it is of great interest to study the variability between two occidental commercial varieties from different botanical groups, since this has not yet been thoroughly exploited through linkage mapping studies. Association studies using accession panels is another approach that has recently been shown to have the potential for characterizing important agronomic traits in melon [11, 12].

In addition to the above-mentioned genetic tools, diverse genomic resources have been developed in melon during the past years. Melon is a diploid species with a small genome (450 Mb) and 12 chromosomes (2n = 24). The use of genomic resources to better understand fruit morphology and quality has been facilitated by the availability of a reference genome [13] and the rapid advances in Next-Generation Sequencing (NGS) technologies, such as RNA-seq [9] and Genotyping-By-Sequencing (GBS) [11, 14, 15]. The GBS strategy is based on the reduction of genome complexity before sequencing, generally through restriction enzyme digestion; only a low percentage of the genome is sequenced but the fragments are normally well distributed across the genome [16]. The GBS approach has been widely used in many species [17–20] due to its simplicity, effectiveness and low-cost when compared to other high-throughput genotyping techniques. The availability of high numbers of SNPs has increased the precision of Quantitative Trait Loci (QTL) mapping. Linkage maps have shown their effectiveness as a tool to study the genetic architecture of both monogenic and complex traits [21]. Recently, high-density maps using hundreds [22] to thousands of markers [10, 14, 15] have been constructed for QTL mapping of fruit traits. It has been demonstrated that a higher SNP density substantially increases the QTL mapping potential, affecting both the detection and the resolution of QTLs [10, 15].

One of the most important aspects for the market is fruit quality. Fruits from the *cantalupensis* group are usually defined by medium fruit weight, round shape, climacteric ripening, orange flesh and white, ribbed and netted rind. In contrast, *inodorus* melons are characterized by non-climacteric ripening, high sugar content, being generally large and elliptical, with green, mottled and smooth rind without ribs nor vein tracts [23]. Several bi-parental mapping and association analyses have been performed for most of these traits, which have been integrated and reviewed by [21]. Some of these traits seem to be under monogenic or oligogenic control [24], such as flesh and rind color, sutures and ribs. However, the most relevant traits related to fruit quality, such as sugar content, fruit size and shape and climacteric ripening are complex and polygenic [10, 25–27]. Extensive research has been done to dissect the genetic control of these traits, but they have been generally limited to crosses between exotic and cultivated material types. Even though they are very valuable, the introduction of exotic alleles in breeding programs is complicated due to linkage drag, with a high cost to remove undesired regions [28]. However, the variation between phylogenetically close but phenotypically different commercial cultivars has not been exploited previously, and can offer new tools easily implemented in breeding programs. The aim of this study was to identify QTLs and major loci related with fruit quality in narrow genomic intervals, using a high-density genetic map obtained with a RIL population from a *cantalupensis* x *inodorus* cross.

## Results

### Phenotyping of the RIL population

The RIL population and the parental lines were evaluated during the summers of 2015 (blocks T1-T3) and 2016 (blocks T4-T5). Several interesting traits related with fruit quality and morphology segregated in the population. Some of these were considered as qualitative (Table 1), although some variation in intensity was observed for MOT, ECOL and YELL. These traits were evaluated for their segregation ratio in the RIL population (Table 1). A segregation of 1:1, expected for a monogenic trait, was observed for ECOL, where the white color of Ved was dominant over green. For MOT, the segregation showed a deviation from the 1:1 expected for a monogenic trait, and the mottled pattern of PS was dominant over its absence. For YELL, a segregation of 3:1 (yellow: not yellow) was observed, in accordance with a dominant epistasis system, where presence of the yellowing allele was dominant.

**Table 1:** Mapping of qualitative traits in the “Védrantais” x “Piel de Sapo” Recombinant Inbred Line population

Trait	PS	Ved	Hyb	Expected segregation	χ^2^	Map position (cM)	Interval^a^	Gene (reference)
External color of immature fruit (ECOL)	Green	White	White	1:1	1.39 ns	39.8	chr07_2707033-chr07_4345823	*Wi* [44]
Mottled rind (MOT)	Yes	No	Yes	1:1	6.22*	127.7	chr02_26206397- end of chr02	*Mt-2* [45]
Yellowing of mature rind (YELL)	Yes	No	Yes	3:1	1.38 ns	34.1	chr10_3152004-chr10_4144573	*CmKFB* [46]
						125.1	chr05_28951742-chr05_29246933	This work

^a^According to version v3.6.1 of melon reference genome

**Fig. 1:**
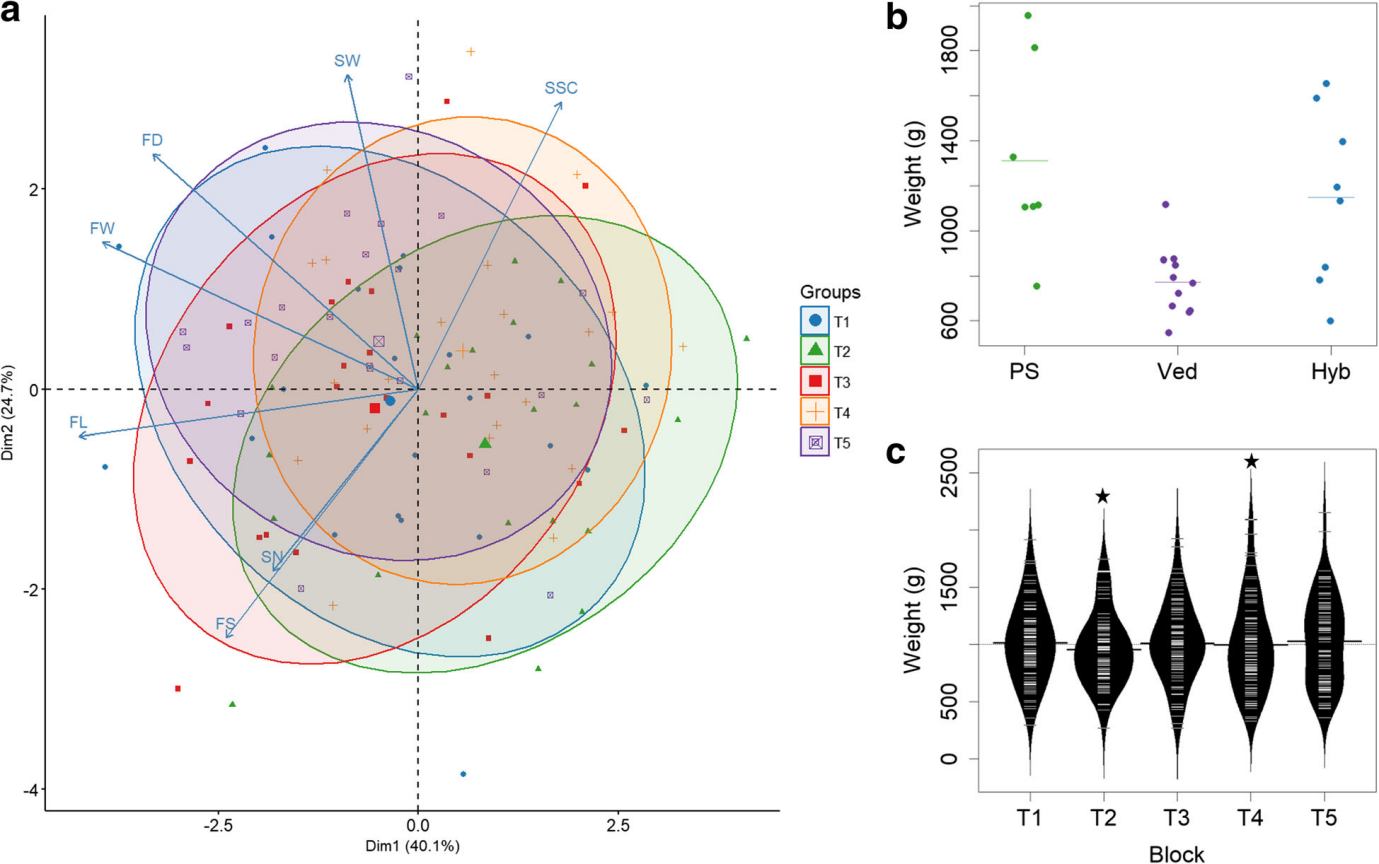
Phenotypic data in the RIL population and the parental lines. **a** PCA showing the similarities between blocks T1 to T5. **b** Distribution of fruit weight in the parental lines, merging data from all blocks. Each dot corresponds to an observation in any of the five blocks T1-T5. The mean for each line is shown with a horizontal line. **c** Distribution of fruit weight in the RIL population in each block T1-T5; black stars indicate significant deviations from normality

The phenotypic values for the quantitative traits are shown in Table 2. In each block, we included the parental lines (Ved, PS) and the Hyb as controls. As an example, fruit weight was lower and quite stable in Ved (771 ± 156 g) when compared to PS and Hyb (1311 ± 428 and 1148 ± 387 g, respectively), with some individuals doubling the weight but showing a higher dispersion (Fig. 1b). The dispersion can be observed in the standard deviation, which is high in complex traits with low heritability (e.g. SSC, FW) and low in more stable traits (e.g. FS) (Table 2, Additional file 1: Figure S1). Transgressive segregation was observed for all traits analyzed.

**Table 2:** Mean and standard deviation of the parental lines and mean and range in the Recombinant Inbred Line population for each quantitative trait

Class	Trait (unit)	Parental lines			RIL population	
		PS	Ved	Hyb	Mean	Range
Fruit quality	SSC (°Brix)	11.8 ± 1.3	10.7 ± 0.8	10.6 ± 1.7	10.4	5.6–14.0
Fruit morphology	Weight (FW) (g)	1311 ± 428	771 ± 156	1148 ± 387	994	345–1763
	Diameter (FD) (cm)	13.0 ± 1.3	11.7 ± 0.6	12.8 ± 1.3	12.0	8.3–14.8
	Shape (FS)	1.36 ± 0.21	1.02 ± 0.22	1.07 ± 0.06	1.2	0.9–1.6
	Length (FL) (cm)	17.8 ± 3.5	12.0 ± 2.8	13.8 ± 2.1	14.0	9.5–19.3
	Perimeter (FP) (cm)	51.5 ± 7.3	39.8 ± 4.9	45.1 ± 5.9	44.0	30.7–53.6
Flesh color	Carotenoid content (CAR) (μg/gFW)^a^	0.7 ± 0.2	18.4 ± 5.6	10.9 ± 1.4	8.8	0.4–30.6
Seed morphology	Seed weight (SW) (mg)	31 ± 4	30 ± 3	37 ± 9	32	18–45
	Seed number (SN)	249 ± 114	324 ± 108	408 ± 194	293	67–499

^a^Only blocks T1, T2 and T3 were analyzed

The distribution of the data for each trait and block was represented with beanplots (Fig. 1c, Additional file 2: Figure S2). The distribution was normal in all blocks for SSC, FL and FS but for FW, FD, FP, SW and SN the deviation from normal was significant in at least one block. CAR was not normally distributed in any of the three blocks analyzed, with more individuals having high-carotenoid content values (> 5 μg/g FW) than intermediate values.

**Fig. 2:**
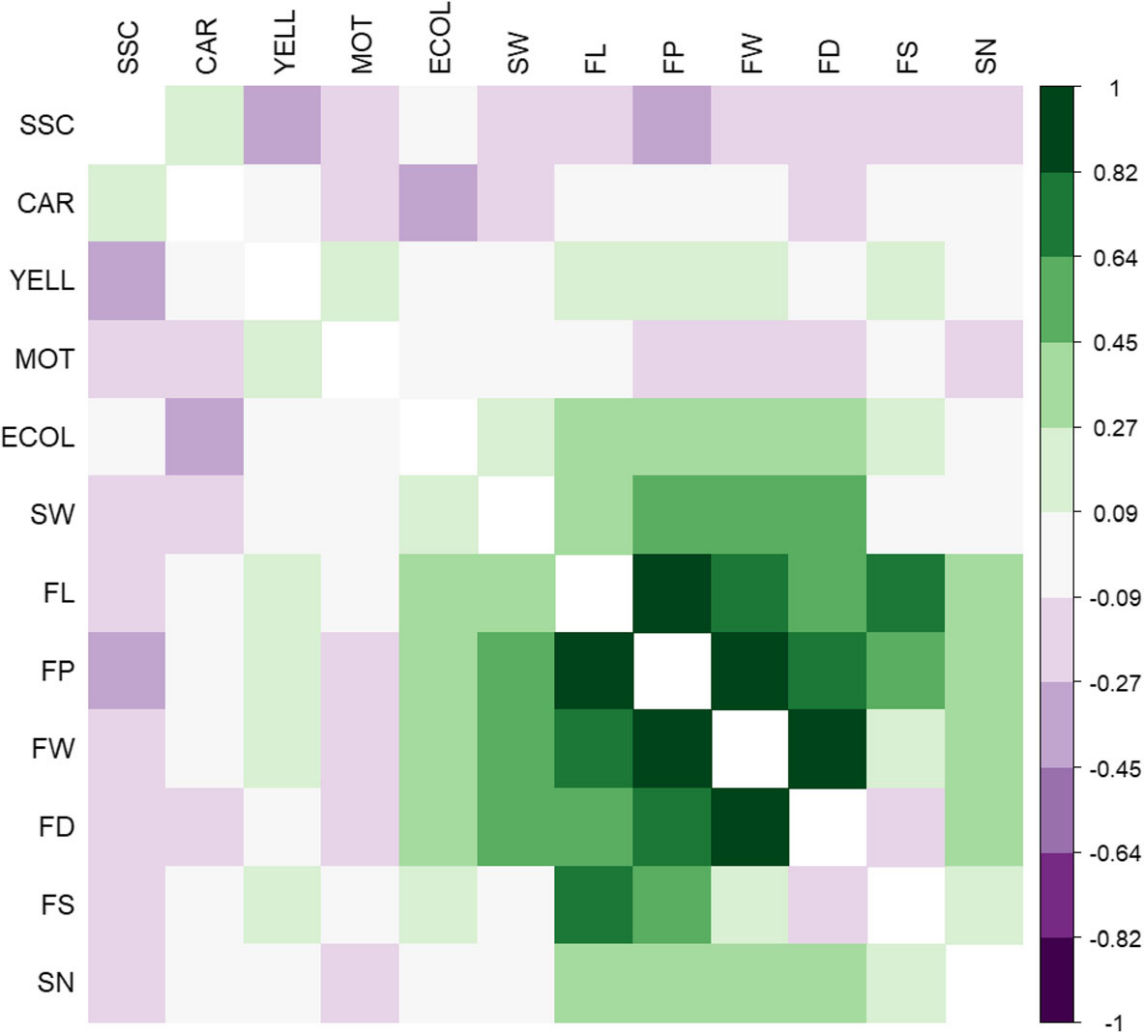
Correlation matrix between the measured traits. The scale represent the values of Pearson coefficient between the traits using the mean value across the blocks for each variable

The correlations between traits are presented in Fig. 2. There was a clear relationship within morphometric measurements. As expected, correlation between fruit dimensions (FL, FD and FP) and FW was strong and positive. The correlation between FS and FL was higher than with FD, implying that length is the major determinant of fruit shape in this population. A positive correlation was detected between seed (SN, SW) and some fruit morphometric traits (FP, FW, FD). ECOL correlated negatively with CAR and positively with FL, FP, FW and FD. YELL negatively correlated with SSC.

### Construction of a genetic linkage map through genotyping-by-sequencing

Sequencing of 91 GBS libraries for the 89 RILs and the two parental lines yielded about 230 million raw reads, corresponding to an average of 2.5 million reads/sample. About 86% of the reads were successfully mapped onto the melon genome (version 3.6.1). A total of 125,465 raw GBS-polymorphisms were called with Fast-GBS. However, about 80% of them were removed due to lack of agreement when compared to the variants from the published re-sequencing data of Ved and PS [36]. The remaining 24,988 pre-filtered variants were further reduced by applying additional filtering criteria (see Material and Methods). In particular, about 50% of the variants were filtered out due to a more restrictive missing value threshold imposed (MV ≤ 60%) and about 20% due to the other criteria imposed (at least one homozygous variant for marker, global quality > 100, only bi-allelic variants). Among the 5944 variants retained, 9.8% were INDEL and 90.2% were SNPs, supported by an average coverage of 17.89. An average of 492 variants per chromosome was detected and chromosome six harbored the highest number (Table 3). A high correlation was observed between the number of variants per chromosome and their physical size. This highlighted that the variants were well distributed and quite uniformly covered all the chromosomes. The distribution of the markers along the 12 melon chromosomes and the unassembled scaffolds (chromosome 0) is given in Table S1a (Additional file 3). A further manual refinement of the marker dataset was carried out to ensure high reliability for the genetic map construction and the QTL mapping analysis, discarding 1056 markers (Additional file 3: Table S1b and Table 3). The 4888 retained GBS-markers were used to build individual bins (Additional file 3: Table S1c). Excluding the bins differing only because of the presence of heterozygous variants, we obtained a total of 824 GBS-derived bins to build the genetic linkage map.

**Table 3:** Variants from the SNP calling and characteristics of the genetic map by chromosome

Chr^a^	Number of variants					Genetic distance (cM)	Total physical distance (bp)^c^	Covered physical distance (bp)^d^	Recombination rate (cM/Mb)
	Raw data	Pre-filtered	Filtered	Genetic map	N° bins				
1	6584	2087	417	360	63	124.6	37,037,532	36,657,204	3.40
2	9643	2511	616	510	71	127.7	27,064,691	26,042,194	4.90
3	12,136	2663	622	508	81	122.6	31,666,927	31,095,866	3.94
4	15,054	2122	534	440	97	156.9	34,318,044	33,448,353	4.69
5	9007	1754	466	365	70	125.1	29,324,171	28,833,706	4.34
6	9067	2575	638	501	79	152.9	38,297,372	37,423,280	4.09
7	13,792	2062	539	460	78	130.9	28,958,359	28,560,617	4.58
8	8242	2281	519	400	67	129.2	34,765,488	32,947,662	3.92
9	6157	1716	419	369	54	109.5	25,243,276	24,844,222	4.41
10	10,363	1776	383	319	45	103.2	26,663,822	23,388,534	4.41
11	14,194	1801	380	326	52	24 + 109^b^	34,457,057	33,905,267	3.92
12	11,226	1640	360	310	58	103.8	27,563,660	26,974,440	3.85
0	–	–	51	20	9	–	–	–	–
Total	125,465	24,988	5944	4888	824	1519.4	375,360,399	367,540,170	–

^a^Melon chromosome

^b^Chromosome XI is divided in two linkage groups

^c^Version 3.6.1 of the melon genome

^d^Subtraction between the first and the last positions covered by markers in the genetic map

**Fig. 3:**
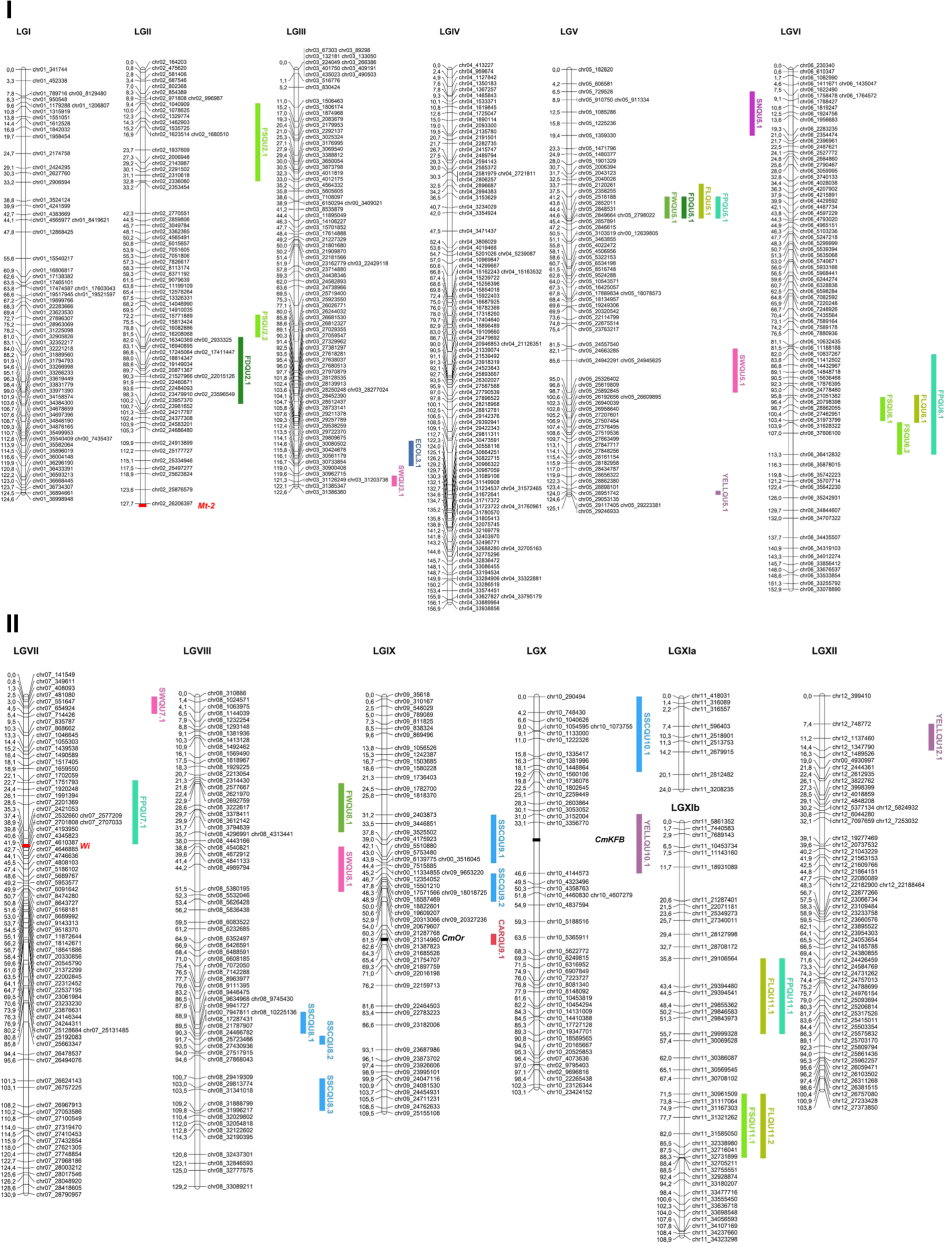
Genetic map containing detected QTLs and major loci. Major loci *Mt-2* (MOT) and *Wi* (ECOL) are placed in their physical position, indicated with a red line. Cloned genes *CmOr* and *CmKFB* are placed in their physical position, indicated with a black line. QTLs are represented as colored bars, using a 1-LOD confidence interval. Green tones for morphological QTLs (FP, FW, FS, FL and FD), pink tones for seed traits (SN and SW), dark blue for ECOL, light blue for SSC, red for CAR and purple for YELL

The genetic distance, the covered physical distance and the recombination rate of the genetic map are presented in Table 3. The map covered 1519.4 cM, distributed in 13 linkage groups (LG) (Fig. 3). Two of them belong to chromosome XI, which was split in two linkage groups LG XIa and LG XIb. The largest LG, 156.9 cM, was LG IV and the smallest one, 103.2 cM, LG X. In terms of physical distance, we calculated the covered region for each chromosome as the difference between the physical positions of the last and the first markers in the LG. The map covered 97% of the melon genome. LG I had the most coverage, with 98.97% of the physical sequence covered by markers, and LG X the least, with coverage of only 87.72% of the sequence represented in the genetic map.

In the genetic map we included nine bins that mapped to chromosome 0, which may help in anchoring additional scaffolds to pseudomolecules. These bins belong to LG I, LG II, LG III, LG V, LG VIII, LG IX and LG XII. We also detected a few inconsistencies between the physical and the genetic map: three bins from chromosomes 2 and 7 according to their physical position were inserted in LG X (not shown).

### Mapping of major loci and QTLs

#### Qualitative traits

**Fig. 4:**
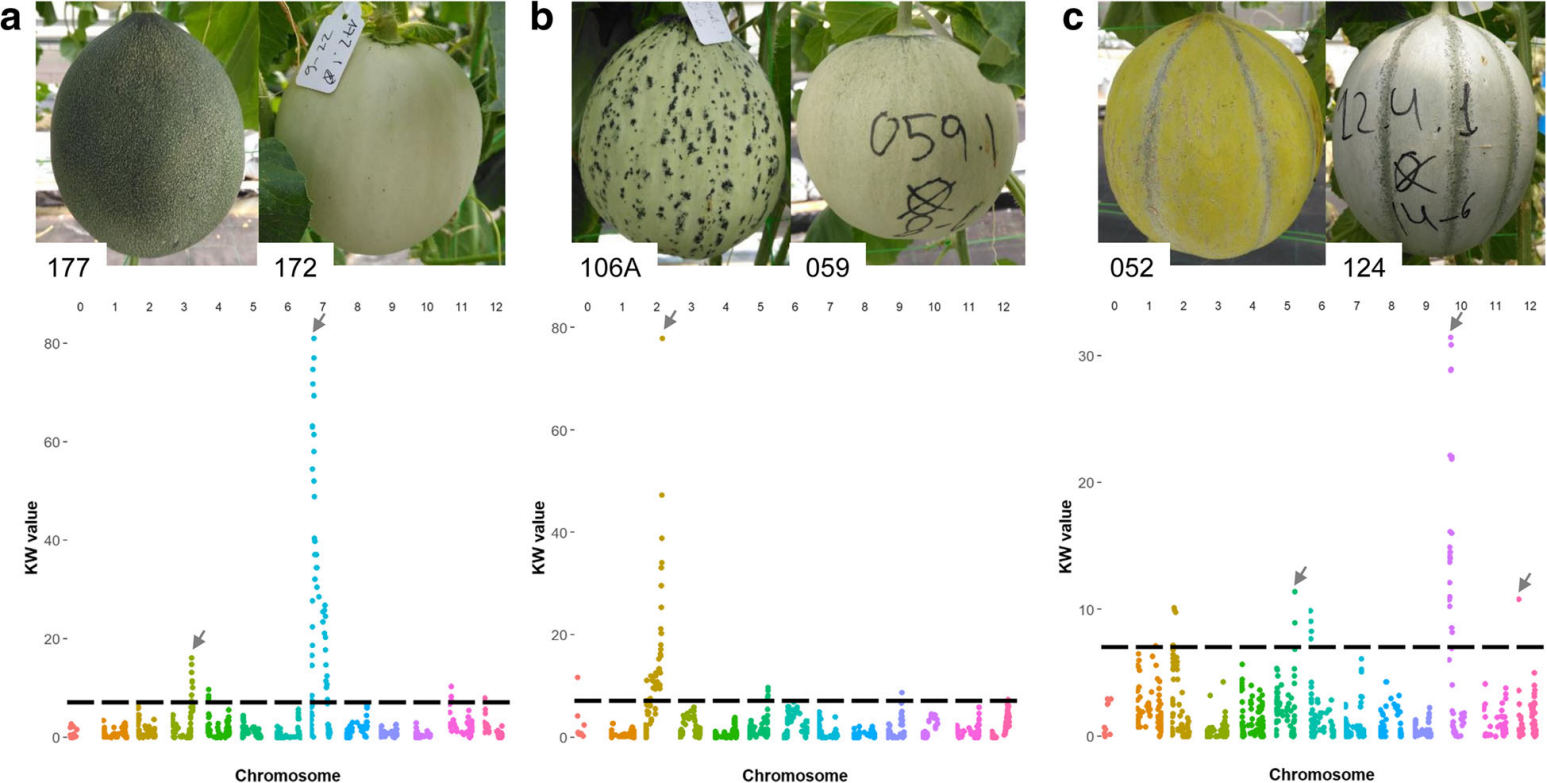
Kruskal-Wallis (KW) statistics test (significant threshold for *p* < 0,01) and photos of fruits showing the two observed phenotypes for ECOL, MOT and YELL. Grey arrows indicate the most significant values. Chromosomes (0 to 12 from left to right) are represented with different colors. **a** External color of immature fruit (ECOL) (RIL 177 green and RIL 172 white). **b** Mottled rind (MOT) (RIL 106A presence and RIL 059 absence). **c** Yellowing of mature rind (YELL) (RIL 052 presence and RIL 124 absence)

For two of the three qualitative traits studied in our RIL population, MOT and ECOL, we detected one major locus controlling the phenotype in LGs II and VII, respectively. In the case of YELL, two minor QTLs in addition to a major locus were observed. In Fig. 4, we show the phenotypical differences between the two categorical classes for each trait and the association between the markers and the phenotype using the non-parametric KW test. In all cases, interval mapping was used to confirm that the results were consistent using both methodologies.

According to the segregation data, ECOL showed monogenic inheritance. This hypothesis was confirmed with the mapping experiments. The gene conferring the external color of immature fruit was located in LG VII, with a KW value of 81.02 at position chr07_4193950 (Fig. 4a). In the interval mapping, a major QTL with maximum LOD of 72.80 at 39.8 cM in LG VII, corresponding to the same physical position as KW, explained 97.7% of the variance and was delimited in a region of 1.6 Mb (Table 1). We detected a second QTL *ECOLQU3.1* with a significant LOD score at 113.4 cM in LG III (Table 4), with an additive effect of 0.2 (greener skin when the Ved allele was present); this QTL can also be seen in Fig. 4a, although with a lower KW value.

**Table 4:** QTL analysis for the traits evaluated. QTLs with LOD > 2.5 using the mean of five blocks (maximum LOD in each block T1-T5 is also annotated)

Trait	QTL ID	LOD	R^2^	Additive effect^a^	Chr	Genetic position (cM)	Physical position^b^ (pb)	Flanking marker 1 respect 1-LOD CI	Flanking marker 2 respect to 1-LOD CI	LOD T1^c^	LOD T2^c^	LOD T3^c^	LOD T4^c^	LOD T5^c^
SSC	*SSCQU8.1*	9.96	40.3	−1.26	8	86.49	9,634,968	chr08_9446475	chr08_17287431	**6.3**	**8.0**	2.7	**5.8**	**4.4**
	*SSCQU8.2*	9.83	39.9	−1.27	8	90.28	2,446,682	chr08_21787907	chr08_25723466	**5.7**	**8.9**	**3.0**	**5.9**	**4.7**
	*SSCQU8.3*	10.76	42.7	−1.3	8	102.66	29,813,774	chr08_29419309	chr08_31888799	**8.2**	**6.2**	**3.8**	**6.9**	**5.9**
	*SSCQU9.1*	2.78	13.4	0.711	9	33.49	3,446,851	chr09_2403873	chr09_6139775	1.6	**4.1**	1.4	2.7	2.5
	*SSCQU9.2*	2.57	12.5	0.69	9	49.89	18,822,601	chr09_12354052	chr09_20679607	0.8	**4.6**	1.5	2.5	2.7
	*SSCQU10.1*	3.3	15.7	0.77	10	18.06	1,448,864	chr10_290494	chr10_1736076	1.5	2.8	1.5	2.6	2.6
FW	*FWQU5.1*	6.42	28.3	153.27	5	40.51	2,516,188	chr05_2356255	chr05_2852011	6.7	2.1	2.3	**3.0**	2.2
	*FWQU8.1*	2.51	12.2	−92.38	8	31.73	3,794,839	chr08_2692759	chr08_4296991	0.8	0.3	1.7	2.5	2.8
FD	*FDQU2.1*	3.29	15.6	0.47	2	88.58	19,149,034	chr02_16082886	chr02_23479910	1.4	1.4	1.7	**3.2**	2.4
	*FDQU5.1*	4.92	22.5	0.6	5	41.22	2,516,188	chr05_2356255	chr05_2852011	**5.5**	1.8	2.3	2.0	1.5
FS	*FSQU2.1*	2.96	14.2	−0.061	2	30.07	2,291,502	chr02_1078625	chr02_2336060	2.6	**3.0**	2.4	**3.6**	0.1
	*FSQU2.2*	3.18	15.2	−0.064	2	75.52	15,813,424	chr02_15771889	chr02_16082886	**4.5**	2.0	0.3	1.5	1.4
	*FSQU6.1*	7.7	32.9	−0.092	6	99.72	27,462,954	chr06_20798398	chr06_31973799	**4.1**	**4.9**	**6.9**	2.5	**5.7**
	*FSQU6.2*	6.96	30.2	−0.087	6	106.99	37,606,100	chr06_31628322	chr06_36412832	**4.3**	**5.0**	**5.6**	2.9	**3.4**
	*FSQU11.1*	3.34	15.9	−0.064	11b	81.68	31,585,050	chr11_30961509	chr11_32731899	2.1	2.8	**4.8**	2.1	1.1
FL	*FLQU5.1*	5.29	24	1.16	5	40.51	2,516,188	chr05_2120261	chr05_2852011	**5.1**	2.3	2.0	**3.8**	2.9
	*FLQU6.1*	5.78	25.9	−1.1	6	100.43	27,462,951	chr06_21051362	chr06_31628322	**3.2**	**4.5**	**3.8**	2.5	**4.6**
	*FLQU11.1*	3.79	17.8	−0.96	11b	47.46	29,855,362	chr11_29106564	chr11_29999328	**3.7**	2.5	1.7	**3.1**	1.8
	*FLQU11.2*	2.67	12.9	−0.81	11b	80.68	31,585,050	chr11_30961509	chr11_32755551	**4.7**	2.8	**4.4**	0.6	0.2
FP	*FPQU5.1*	6.73	29.4	2.95	5	40.51	2,516,188	chr05_2356255	chr05_2852011	**7.7**	2.8	2.6	**3.2**	2.5
	*FPQU6.1*	2.75	13.3	−1.81	6	100.43	27,462,951	chr06_11412502	chr06_36412832	1.5	**3.2**	1.6	1.7	2.0
	*FPQU7.1*	2.54	12.3	−1.83	7	29.47	2,201,369	chr07_1702059	chr07_2701808	1.0	1.8	1.1	2.4	1.7
	*FPQU11.1*	3.27	15.6	−2.03	11b	42.79	29,394,480	chr11_29106564	chr11_29999328	**3.5**	2.7	1.1	**3.0**	1.1
YELL	*YELLQU5.1*	3.15	15.1	−0.15	5	125.12	29,117,405	chr05_28951742	chr05_29246933	**3.8**	2.8	1.4	2.5	1.9
	*YELLQU10.1*	8.79	36.6	−0.25	10	34.07	3,356,770	chr10_3152004	chr10_4144573	**8.1**	**5.9**	**4.2**	**6.6**	**6.7**
	*YELLQU12.1*	2.96	14.2	0.15	12	11.21	1,137,460	chr12_748772	chr12_1347790	1.7	1.6	1.4	2.8	2.4
ECOL	*ECOLQU3.1*	3.49	16.5	0.21	3	113.40	29,722,370	chr03_29257789	chr03_30733854	**3.5** ^**d**^			**3.0** ^**d**^	
CAR	*CARQU9.1*	16.82	58.5	5.6	9	64.32	21,685,526	chr09_21387823	chr09_21754707	**12.7**	**11.6**	**13.6**	–	–
SN	*SNQU5.1*	2.73	13.2	33.43	5	14.46	1,225,236	chr05_729528	chr05_1359330	2.1	0.5	0.3	2.1	0.4
SW	*SWQU3.1*	4.11	19.2	2.7	3	122.11	31,385,347	chr03_30962715	chr03_31386360	2.4	1.6	0.3	2.7	1.7
	*SWQU5.1*	4.3	19.9	2.9	5	87.63	24,945,625	chr05_24663285	chr05_25326402	**3.0**	2.6	**3.2**	2.4	1.2
	*SWQU7.1*	2.91	14.0	−2.2	7	0.78	349,611	chr07_141549	chr07_654924	0.9	1.4	1.5	0.9	2.0
	*SWQU8.1*	4.29	19.9	−2.7	8	43.35	4,989,794	chr08_4672912	chr08_5308195	0.9	**6.9**	1.9	**3.1**	1.2

^a^Additive effect of the Ved allele

^b^Physical position is relative to the melon genome sequence v3.6.1

^c^Bold and underlined font for LOD scores above 3, underlined font for LOD scores between 2.5–3

^d^LOD scores for 2015 and 2016, respectively

The evaluation of MOT was difficult in some fruits. The allele that confers the mottled rind is from PS, but it is not easily detected due to the dark green color of the PS rind, which masks the darker spots (Additional file 4: Figure S3a). In contrast to the striking appearance of dark spots in melons with white rind (Fig. 4b). Although the segregation did not follow the expected 1:1 ratio for a monogenic trait (Table 1), the KW test and interval mapping clearly showed a major locus in the distal part of LG II (Fig. 4b). In fact, after analyzing the segregation of the markers in this region of the genetic map, we observed a segregation distortion (χ^2^ = 6.40 in the closest marker). The major locus is at the end of the LG, with chr02_26206397 the last marker of the linkage group and the most associated to the phenotype, with a LOD = 63.90 in interval mapping. A region in the melon genome of approximately 0.8 Mb distal to chr02_26206397, not covered by markers in the genetic map, was considered in the QTL interval (Table 1).

Another qualitative trait evaluated was the yellowing of mature rind (YELL). As with the mottled rind, the yellow allele comes from PS. It is partially masked by the dark green rind color, but has a different tonality, leading to a greyish color which is visible when the yellow allele is absent (Additional file 4: Figure S3b). The observed segregation suggested the hypothesis of two genes under dominant epistasis. The first and most important gene is in LG X, in a region of approximately 1 Mb (Table 1 and Table 4) and was detected in the KW test (Fig. 4c) and in interval mapping with a LOD = 8.79. Two other QTLs were detected: *YELLQU5.1* in LG V, explaining 15.1% of the variance with the Ved allele decreasing the yellow color; and *YELLQU12.1* in LG XII, explaining 14.2% of the variance with the Ved allele increasing the yellow color. Both QTLs can also be observed in Fig. 4a, although with lower KW values.

#### Quantitative traits

QTL mapping was performed using the mean of five blocks and using each block individually (T1-T5) (Table 4, Fig. 3). A QTL was considered significant with a LOD score higher than 2.5 in the mapping analysis that considered the mean phenotypic values. We also show the LOD for the same QTL/position in the individual blocks. Thirty-three significant QTLs were detected for the 11 measured traits. The level of consistency between blocks depended on the trait and the significance of the QTL. Although some QTLs seemed to be dependent on the year, for example *YELLQU12.1*, with higher LOD scores in both 2016 blocks than in the three 2015 blocks, although this effect was not general.

##### Fruit quality traits

We evaluated SSC, an important trait concerning fruit quality in melon. Both parental lines are commercial types and the SSC is acceptable, but in PS it is slightly higher than in Ved. In our evaluations, it ranged from 10.5 to 13.1 °Brix in PS and from 9.9 to 11.5 °Brix in Ved. The hybrid was similar to Ved (Table 2 and Additional file 1: Figure S1).

Six significant QTLs were detected for SSC (Table 4). Among them, *SSCQU8.3* was the most consistent, with a LOD score above 3.5 in all experiments; it explained 42.7% of the variance and the Ved allele presented reduced 1.3 °Brix. The QTL is located in LG VIII around 102.66 cM, in an interval of 2.5 Mb.

Although SSC content is higher in PS than in Ved, in three out of six QTLs the Ved allele had a positive effect, explaining the transgressive segregation observed in the RIL population. The percentage of variance explained by each of them was around 13% and the additive effect of the Ved allele was 0.7 °Brix.

##### Fruit morphology traits

We evaluated five traits related with fruit morphology: weight, diameter, shape, length and perimeter. In total, we detected 17 QTLs; some were exclusive for a single trait (e.g. *FSQU2.1*) and others co-localized for several morphological traits (e.g. *FWQU5.1*, *FDQU5.1*, *FLQU5.1* and *FPQU5.1*) (Fig. 3, Table 4).

The most significant QTL for FW was *FWQU5.1* in LG V, explaining 28.3% of the variance; the allele of Ved increased average fruit weight to 153.27 g. It was detected in the mean analysis with a LOD score of 6.42 and in T1 and T4 with LOD ≥ 3. A QTL in the same interval was also detected with high LOD scores for FD, FL and FP, indicating a major effect on fruit size in this region. The best resolution for this QTL was obtained for *FWQU5.1*, *FDQU5.1* and *FPQU5.1*, which delimited it to a 500-kb interval.

*FLQU6.1* in position 100.4 cM of LG VI, had a LOD score of 5.78 in the mean analysis and was significant in all blocks (T1-T5). It explained 25.9% of the variance and the Ved allele decreased the length of the fruit by 1.1 cm. The QTL was located in an interval of 10.6 Mb in the centromeric region of the chromosome.

Concerning FS, we describe five QTLs and in all cases, the Ved allele decreased the shape index to produce rounder fruits. A QTL co-localizing with *FLQU6.1*, *FSQU6.1*, was the most significant and consistent. *FSQU2.1*, in a region of approximately 1.3 Mb in LG II, did not co-localize with any other morphological trait-associated QTL.

##### Flesh color traits

Although the gene determining orange flesh color, *CmOr*, has already been described by [47], we decided to measure total carotenoids content in flesh of ripe fruit. We observed a transgressive segregation (Table 2), finding almost double the total carotenoids as the mean of Ved in some RILs. The QTL mapping revealed just one major QTL in LG IX with a LOD = 16.82 explaining 58% of the variance (*CARQU9.1*, Fig. 3). We did not detect any other minor QTL for this trait.

##### Seed traits

Although the mean values for the parental lines were similar, we detected four significant QTLs for seed weight (Table 4, Fig. 3). None of the QTLs for seed weight co-localized with fruit morphology QTLs. The most significant was *SWQU8.1*, with a LOD score of 4.29, explaining 19.9% of the variance in the RIL population. The Ved allele diminished seed weight. *SWQU8.1* was located in a region of 636 kb. A single QTL *SNQU5.1* for seed number was detected in LG V, but with a lower LOD score.

## Discussion

### The GBS approach applied in a biparental RIL population is highly effective for QTL mapping studies

Understanding the genetic control of important agronomic traits has been a challenge over the last few decades. Different strategies have shown their effectiveness, but the most used is the QTL mapping approach. Type and size of the population and map density are the main limiting factors for detecting QTLs and their resolution. RIL populations present some advantages: the lines are fixed, so multiple evaluations in different years or environments are possible; each individual has potentially suffered multiple recombination events, increasing the mapping resolution; and the development of this type of population is simple and of low cost using a single-seed descent method without need for intermediate genotyping [48].

Until recently, the main limiting factor, in terms of work and cost, was marker discovery and genotyping. The first genetic maps used during the eighties and nineties generally included from tens to a few hundred markers, mainly isoenzymes and RFLPs [49–51]. Due to the fast development of sequencing technologies and bioinformatics, genotyping is becoming more and more affordable and accessible to the scientific community. A reference genome sequence has already been published for many important crops, including maize [52], rice [53] and tomato [54], among others, facilitating the use of high-throughput genotyping methods based on NGS. The GBS strategy is by far the most widespread technique for high-throughput genotyping, allowing simultaneous variant calling and genotyping for thousands of SNPs and INDELs, and without the need for a reference genome. In melon, GBS has recently been used to characterize collections of accessions [11, 14, 55] and biparental populations [14, 15]. The number of variants we obtained (24,988 SNPs and INDELs) was comparable to those obtained in these previous studies, ranging from 13,756 to 99,263. Such a divergence in the number of variants is expected, depending on factors such as the diverse origin of the germplasm, the sequencing technology used, the software chosen for variant calling and the filtering criteria applied. Also the possibility to impute or not the missing values could greatly affect the final number of variants. The number of bins found in our Ved x PS linkage map (824) was lower than in previous studies, 1837 [14] and 2493 [15] respectively. This discrepancy was expected since in those studies the founding cross for the RIL population was between *C. melo* spp. *melo* and *C. melo* ssp. *agrestis* accessions, which represent wider diversity in comparison to our population.

Although many QTL mapping studies using less dense linkage maps have confirmed their effectiveness, increasing the number of markers allows full exploitation of the recombination events in the population, improving the resolution of the QTLs. In a RIL population, where multiple meiosis could derive in short bins, this effect could notably increase not only the resolution but also the power of detection, especially for minor QTLs [15]. The power of detection is not comparable among different populations, but the resolution in our QTL mapping had a median QTL confidence interval of 9.42 cM and 0.94 Mb in genetic and physical distances, respectively. These results are comparable with the 4.04 cM and 0.93 Mb obtained in [15] and more precise than in other recent studies using less dense maps, where the QTL genetic confidence interval ranged between 23 cM [5] and 28.6 cM [4].

To validate our QTL mapping results, we used as a proof of concept two fruit quality traits that segregate in our population whose subjacent genes are already known, *CmOr* [47] and *CmKFB* [46] (Additional file 5: Table S2). *CmOr* determines the orange flesh in ripe melon when the dominant allele is present, by inducing the accumulation of β-carotene. We mapped a major QTL for CAR, *CARQU9.1*, at position 21,685,526 in chromosome IX, in a confidence interval of 366.8 Kb containing 47 genes according to the annotation version v4.0 of the melon genome [56] (Table 5). To observe the expression pattern of the candidate genes in this interval we used the atlas expression database Melonet-DB [57], developed using 30 different tissues from the *cantalupensis* variety “Harukei-3”. We could reduce the number of candidate genes to 19 that were expressed in fruit flesh from 20 to 50 DAP, and only three had sequence differences between Ved and PS causing a non-synonymous amino acid change. *CmOr* was included in this final group, and the maximum LOD position of the QTL was within this gene (*MELO3C005449*, coordinates 21,683,406-21,690,712). *CmKFB* controls the biosynthesis of flavonoids in ripe melon rind, conferring the yellow external color typical of “CanaryC yellow” melons. We detected a major QTL for this trait in our RIL population, *YELLQU10.1*, at position 3,356,770 in chromosome X. The confidence interval of 992 Kb contained 156 genes (Table 5), of which 33 presented variations in our population causing a non-synonymous amino acid change. *CmKFB* was among them and the maximum LOD position was located approximately 100 kb upstream of this gene (*MELO3C011980*, coordinates 3,475,283-3,476,416).

**Table 5:** Genomic intervals containing the identified QTLs for each trait and number of annotated genes in each interval

Trait	Gene/QTL ID	QTL interval (cM)	QTL interval (pb)	Number of annotated genes^a^
SSC	*SSCQU8.1*	5.64	7,840,956	389
	*SSCQU8.2*	2.23	3,935,559	195
	*SSCQU8.3*	8.55	2,469,490	123
	*SSCQU9.1*	12.68	3,735,902	290
	*SSCQU9.2*	7.36	8,325,555	525
	*SSCQU10.1*	19.75	1,445,582	231
FW	*FWQU5.1*	6.28	495,756	48
	*FWQU8.1*	12.76	1,604,232	220
FD	*FDQU2.1*	19.51	7,397,024	501
	*FDQU5.1*	6.28	495,756	48
FS	*FSQU2.1*	22.77	1,257,435	136
	*FSQU2.2*	6.55	310,997	34
	*FSQU6.1*	6.98	11,175,401	545
	*FSQU6.2*	9.34	4,784,510	432
	*FSQU11.1*	16.77	1,770,390	221
FL	*FLQU5.1*	10.10	731,750	82
	*FLQU6.1*	8.11	10,576,960	505
	*FLQU11.1*	19.96	892,764	108
	*FLQU11.2*	16.99	1,794,042	224
FP	*FPQU5.1*	6.28	495,756	48
	*FPQU6.1*	29.43	25,000,330^a^	1444
	*FPQU7.1*	16.80	999,749	149
	*FPQU11.1*	19.96	892,764	108
YELL	*YELLQU5.1*	1.11	295,191	54
	*YELLQU10.1* (*CmKFB*)	15.61	992,569	156
	*YELLQU12.1*	7.00	599,018	65
MOT	*Mt-2*	–	858,294	139
ECOL	*ECOLQU3.1*	7.37	1,476,065	238
	*Wi*	–	308,385 + 271,774^b^	41 + 19
CAR	*CARQU9.1* (*CmOr*)	2.77	366,884	47
SN	*SNQU5.1*	12.92	629,802	76
SW	*SWQU3.1*	2.99	423,645	73
	*SWQU5.1*	12.96	663,117	85
	*SWQU7.1*	4.48	513,375	90
	*SWQU8.1*	11.92	635,283	94
Median		9.42	942,667	130

^a^Annotation version v4.0 of the melon genome (http://www.melonomics.net)

^b^An inconsistency between the physical and the genetic map exists in this region

### Deciphering the genetic architecture of fruit quality and domestication traits in melon

Deciphering the genetic control of important traits in crops is one of the main objectives of modern research in agriculture. The knowledge of the responsible genes would offer the opportunity to explore the functional mechanisms that control phenotypes, allowing the search and study of allelic diversity of cultivars and accessions and ultimately to modify crop behavior. As a first step, our work identified major loci and QTLs involved in important traits in melon.

#### Rind traits

External color of fruit is an important trait concerning fruit quality, since the appearance is one of the main determinants for consumer choice in the market. The phenotype varies depending on the developmental stage and is determined mainly by the accumulation of pigments such as chlorophylls, carotenoids and flavonoids [58]. In our RIL population, at least two major traits control rind color: ECOL, conferring white or green rind in immature fruit, and YELL, determining the yellowing of mature rind, probably involving biosynthesis of flavonoids. Since pigment analyses was not performed, we cannot discount that other factors, such as the exposure of β-carotene after the degradation of chlorophyll due to climacteric ripening, affect the trait.

The external color of immature fruit was previously described by [44] as a monogenic trait named *Wi*, but to our knowledge it has not been mapped. More recently, four loci involved in ECOL have been identified in LGs III, VII, IX and X using two mapping populations derived from PS and PI 161375, suggesting an epistatic interaction between at least some of them [59]. Our RIL population shares one parental (PS) with this study, and we mapped a minor QTL (*ECOLQU3.1*) and a major QTL (*Wi)* in the same chromosomes as *ECOLQC3.5* and *ECOLQC7.2* [59], respectively, however their physical positions do not co-locate (Additional file 5: Table S2). This trait has been characterized in cucumber, identifying a candidate gene, a *two-component Response Regulator-like Protein* (*APRR2*) [60, 61]. We found no *RRP* gene in the confidence interval containing *Wi*, however there was an inconsistency in the genome assembly in this region that could affect this result (Table 5). *ECOLQU3.1*, having a minor effect in comparison to *Wi*, could slightly modify the external color by affecting the same pathway or by another mechanism.

The yellowing of mature rind has been described before, with the flavonoid naringenin chalcone identified as the principal pigment responsible for the yellow color in melon cultivars such as “Noy Amid” [58]. Recently, a Kelch domain-containing F-Box protein coding gene (*CmKFB*) was cloned by [46], showing that this protein is the main regulator of the flavonoid biosynthetic pathway. In addition to naringenin chalcone, other downstream flavonoids have been identified in yellow melon rind. In other species, the complex of MYB-bHLH-WDR transcription factors has been shown to control flavonoid production [62, 63]. The major QTL *YELLQU10.1* interval contains the gene *CmKFB*, as explained above. According to the observed 3:1 segregation for this trait (Table 1), *YELLQU5.1* could act epistatically with *CmKFB*, regulating the biosynthesis or accumulation of naringenin chalcone or other flavonoids. Following this hypothesis, the yellow rind phenotype in our RIL population could be determined by PS alleles in any of the two genes. Within the *YELLQU5.1* interval, which contains 54 annotated genes, we identified *MELO3C004621*, which is described as a “Ectonucleotide pyrophosphatase/phosphodiesterase” and could be involved in the flavonoid pathway based on homology; additionally, this gene is highly expressed in fruit rind during the last stages of development in Melonet-DB [57] and carries a variant that produced an amino acid change in the protein. However, further experiments are necessary to demonstrate the identity of *YELLQU5.1*.

The presence or absence of a mottled pattern also influences the rind appearance; this trait (*Mt-2*) is controlled by a major locus in LG II previously described by [45, 64], probably the same one that was mapped in our population (Fig. 4b). Although this pattern can be observed in other cucurbits, both the genetic control and the physiological mechanism remain unknown. One hypothesis is that the spots correspond to areas of the rind where the chlorophyll content is higher, due to an increased number, size and/or activity of chloroplasts. This hypothesis is supported by the observation of a more intense yellow in spots of mature rinds, when the allele for yellowing is present and the chlorophyll is degraded due to climacteric ripening. *Mt-2* is located in an interval of 858 kb that contains 139 annotated genes, without any candidate gene by functional annotation.

The rind color of fruits in our RIL population should be determined by these three traits and modified by other important aspects of fruit development, such as the type of fruit ripening (climacteric or non-climacteric), where chlorophyll degradation can be involved [65]. As discussed above, we cannot rule out that yellowing of the rind is a consequence of climacteric ripening in some of the fruits from our RIL population. 

#### Soluble solid content

Melon fruit is mainly consumed as a dessert, a high content of sugars being a desired characteristic with special importance in crop improvement. Ved and PS are both commercial varieties on the European market, with medium-high soluble solid content, so we did not expect to find major QTLs for this trait (Table 2).

Three QTLs were found in LG VIII with LOD > 9, between the physical positions 9,446,475 and 31,888,799 bp, explaining around 40% of the variance and the Ved allele decreasing 1.3 °Brix. We detected three clear peaks even increasing the confidence interval of these QTLs (Additional file 6: Figure S4), but it could still be possible that there is a single QTL in this region and the low-LOD regions inside the interval were artefactual. The higher LOD value corresponds to *SSCQU8.3*, delimited in a region of 2.5 Mb that contains 123 genes (Table 5). In other studies, QTLs for SSC have also been detected in LG VIII (Additional file 5: Table S2); [66] found two introgression lines (ILs) in the PS background containing introgressions from the exotic accession PI 161375 that covered the major part of chromosome VIII, including *SSCQU8.3*, with a significantly different SSC content.

Another three QTLs were detected in LG IX and LG X, *SCCQU9.1*, *SSCQU9.2* and *SSCQU10.1*. Although having a lower effect in SSC, they are interesting because in all cases the Ved allele increases sugar content. However, they are unstable, showing significant LOD scores only in T2, T4 and T5. QTLs for SSC in LGs IX and X have been previously described in similar positions to *SSCQU9.2* and *SSCQU10.1* (Additional file 5: Table S2).

#### Fruit morphology

Fruit morphology, including weight, size, length, diameter and shape, are key traits in the domestication process, enabling discrimination between cultivated and wild accessions. Due to their importance, they have been extensively studied in many species, especially in tomato, where several genes have been cloned (reviewed in [26]). Known genes controlling fruit size in tomato are *Cell Number Regulator/FW2.2*, *SlKLUH/FW3.2*, a cytochrome P450 A78 class, and *Cell Size Regulator/FW11.3* [67]. Fruit shape is mainly determined by the combination of different alleles of *FAS*, from the YABBY family; *SUN*, an IQ domain member; *LC*, the homologue of *WUS*, and *OVATE*. In melon, with different populations used for QTL mapping studies, meta-QTLs implicated in fruit morphology have been identified [4, 59, 68], unfortunately, none of the genes responsible for these QTLs have been cloned. All the QTLs described in the present work are supported by previous research that identified QTLs in the same LGs (Additional file 5: Table S2), except *FPQU7.1*. Although the physical positions associated to the QTLs are not always similar, it should be noted that only one marker was used to calculate the position of several QTLs described in Table S2 (Additional file 5), which usually span the major part of the LG. For example, *SC5–2* was described by [7] using an introgression line that covers almost all LG V (0–20,855,850 bp).

A clear transgressive segregation was obtained for fruit weight, from a mean of 345 g in RIL 172 to 1763 in RIL 140 (Additional file 7: Figure S5). Consistently, a QTL explaining 28.3% of the variation, *FWQU5.1*, increased the weight when the Ved allele was present. A QTL in the same chromosome has been previously described using populations developed from a cross between PS and the exotic Korean accession PI 161375 (Additional file 5: Table S2). The 496-kb interval of *FWQU5.1* contains 48 genes, and among them only 23 were expressed following the expected pattern for fruit size regulators (ovary and young fruit) using the atlas expression database Melonet-DB [57]. Five of these genes carried variants in the sequence provoking a non-synonymous change between Ved and PS. One of these genes is *MELO3C014402*, described as FANTASTIC FOUR 2 in the annotation v4.0. These proteins are usually related to meristem development [69] and *Cell Size Regulator*, the gene underlying a recently cloned fruit weight QTL in tomato contains a FANTASTIC FOUR domain [67].

*FLQU6.1* and *FSQU6.1* are in the same region in the centromere of chromosome VI, implying that the decrease in length caused by the Ved allele provokes a decrease in the shape index. They co-localize with a QTL published recently for the same traits [68], mapped in an F_2_ population between PS and the Indian wild accession “Trigonus” and validated using introgression lines. In this case, the PS allele also increases fruit length and the percentage of variance explained is similar, around 20%. The segregation of this QTL in commercial varieties suggests that it is a diversification not a domestication QTL, according to classical definitions.

Possibly, orthologs of the genes that regulate fruit size and shape in tomato could be implicated in the same process in melon, and thus be candidate genes underlying the detected QTLs. In order to evaluate whether they co-localize within the QTL intervals, we identified the potential fruit morphology orthologs in the melon genome (version 3.6.1 and annotation v4.0) (Additional file 8: Table S3A), which resulted in the identification of 89 genes. Twelve of them are contained in the intervals of *FSQU2.1*, *FDQU2.1*, *FSQU6.1*, *FSQU6.2*, *FWQU8.1* and *FSQU11.1* (Additional file 8: Table S3B). Among them, there are four genes (*MELO3C015418*, *MELO3C025343*, *MELO3C013751*, *MELO3C022253*) that showed the expected pattern of expression, being specific for ovaries and young fruit, according to the melon expression atlas [57]. In addition, *MELO3C015418* and *MELO3C025343* carried one and two non-synonymous polymorphisms between the parental lines, so they could be the candidate genes for *FSQU2.1* and *FDQU2.1*, respectively. Additional studies, such as QTL fine mapping and differential expression analysis between Ved and PS, is needed to confirm their involvement in fruit morphology.

#### Seed traits

Seeds are the most valuable part of the fruit in terms of evolution, so both seed weight and number are important fitness traits. Although these traits have been widely studied in crops in which grain is consumed, such as rice [70–74] and soybean [75, 76], in vegetable crops much less is known. Although seed is not consumed in many vegetable crops, it is the product that seed companies commercialize. A higher seed production and better seed quality are interesting traits for both breeders and farmers.

In melon, seed size is considered a domestication trait, since wild accessions have smaller seeds than cultivated melons [3]. The genetic basis of this trait has not been well studied; a recent QTL mapping study, focused on domestication traits, evaluated seed weight, without the identification of any QTL [68], suggesting that a complex inheritance involving several minor QTLs could be the reason.

Surprisingly, in our RIL population, which is founded by two phylogenetically close commercial cultivars, we identified four QTLs for seed weight. SW was positively correlated with FP, FW and FD, but SW QTLs did not co-localize with fruit morphology QTLs (Figs. 2 and 3). Due to the high stability of the trait (Additional file 1: Figure S1), we could map these QTLs in narrow regions of the genome, spanning between 423 and 663 kb, containing, in all cases, less than 100 genes. The stability of seed size has been studied previously in multiple crops, showing that this trait has low dispersion even in different environmental conditions, unlike seed number, which is a very plastic trait [77].

## Conclusions

QTL mapping using the RIL population Ved x PS identified several QTLs and major loci that modify and modulate fruit quality, from the external appearance to the biochemical composition. The location of these QTLs in narrow genomic intervals could facilitate cloning of the underlying genes and their use in breeding programs by marker-assisted selection. The introgression of favorable alleles into breeding lines could be performed easily, since the mapping population was developed from commercial cultivars, avoiding the negative consequences associated to linkage drag when using exotic material as donors. Thanks to the reduced number of annotated genes in some of these intervals, potential candidate genes have been proposed through an integrative strategy that included the analysis of gene expression and the predicted effect of variants using genomics databases.

## Materials and methods

### Plant material

A RIL population was generated from two commercial varieties, “Védrantais” (Ved) (ssp. *melo*, *cantalupensis* group) and “Piel de Sapo” T111 (PS) (ssp. *melo*, *inodorus* group). Ved is a French variety from the group *cantalupensis* that produces medium-size, rounded fruits, with white external coloration when immature and cream after ripening, and with orange flesh. PS is a Spanish variety from the group *inodorus*, with large, elongated and mottled fruits, with dark green rind and white flesh. Both varieties have high soluble solid content since they are accepted in European markets.

The RIL population was developed in greenhouses at Cabrera de Mar (Barcelona) and Caldes de Montbui (Barcelona), through a strategy of single seed descent to F_7_-F_8_, starting from an F_2_ population obtained in 2008. The population was composed of 89 RILs, including 82 RILs in the F_8_ generation and seven in the F_7_. A set of 48 polymorphic SNPs evenly distributed through the melon genome was used to confirm the homozygosity of the RILs, which was higher than 98% (not shown).

The RIL population was grown in Caldes de Montbui (Barcelona) under greenhouse conditions during the summers of 2015 (three blocks T1-T3) and 2016 (two blocks T4-T5). Plants were pruned weekly and each plant was allowed to set only a single fruit. Each block (T1-T5) contained a single individual per RIL and 1–3 individuals of the parental lines (Ved, PS) and the F_1_ (Hyb). Flowers were hand-pollinated and the date was recorded to register the total days of development of the fruit until harvest. According to the type of ripening of each RIL, the harvest date was recorded as follows: (1) the abscission date for climacteric fruits showing abscission, (2) seven days after the first symptom of climacteric ripening (aroma production, chlorophyll degradation or abscission layer formation) for climacteric fruits that did not show abscission and (3) 60 days after pollination for non-climacteric fruits.

Blocks T1-T3 (2015) were grown during the same season but each block was separated by three weeks. Blocks T4-T5 (2016) were grown together. A mean of 8.6 lines per block were not evaluated due to problems related to seed germination, plant disease or fruit set; 60 out of 89 RILs (67%) were evaluated in the five blocks.

### Phenotyping of fruit and seed traits

Fruit quality traits (Table 6) were recorded during the development of the fruit and at harvest. Mottled rind (MOT) and external color of immature fruit (ECOL) were phenotyped as qualitative traits, before onset of ripening, around 20–30 days after pollination (DAP). Data for these traits were merged by year, due to the low variability observed among blocks (> 95% of the RILs showed the same phenotype across blocks).

**Table 6:** Traits evaluated in the “Védrantais” x “Piel de Sapo” Recombinant Inbred Line population

Trait	Code
Soluble solid content	SSC
Fruit weight	FW
Fruit diameter	FD
Fruit shape	FS
Fruit length	FL
Fruit perimeter	FP
Yellowing of mature rind	YELL
Mottled rind	MOT
External color of immature fruit	ECOL
Total carotenoid content	CAR
Seed weight	SW
Seed number	SN

After harvest, fruits were weighed and cut in two longitudinal sections: the first was scanned for morphological analysis using the Tomato Analyzer 3.0 software [29, 30] and the second section was used to measure soluble solid content (SSC) and total carotenoids (CAR). The morphology traits recorded were fruit weight (FW), diameter (FD), length (FL) and perimeter (FP) with the Tomato Analyzer 3.0 software, and shape index (FS) was calculated as the ratio between FL and FD. To measure SSC, four flesh samples of 1 cm diameter per melon were used; the juice was extracted by pressuring the samples against a strainer and analyzed with a digital hand refractometer (Atago Co. Ltd., Tokyo, Japan). Total carotenoid analysis was performed by UV-VIS Spectroscopy [31] as described by [32], using the flesh from the first three blocks (T1-T3). The yellowing of mature rind (YELL) was recorded as a qualitative trait (0 absence, 1 presence) by visual inspection of ripe fruits. Fifteen dried seeds were used to estimate seed weight (SW) and seed number (SN) per fruit.

### Genotyping and linkage map construction

Young leaves from the RIL population and the parental lines Ved and PS were collected during the summer of 2015 and stored at − 80 °C. DNA extraction was following the CTAB protocol [33] with some modifications. Briefly, the isopropanol precipitation was followed by incubating 30 min at 4 °C and centrifuging for 10 min after adding the washing buffer. The extracted DNA was re-suspended in MilliQ water. PicoGreen® dsDNA Assay Kit (Life Technologies) was used according to the manufacturer’s protocol for quantity assessment, and DNA quality was estimated by gel electrophoresis.

GBS was performed at the National Center of Genomic Analyses (CNAG, Barcelona, Spain). *Ape*KI GBS libraries of the 89 RIL and the parental lines (PS and Ved) were prepared at CNAG and sequenced using Illumina HiSeq2000 (2 × 100 bp).

The Fast-GBS pipeline [34] and the melon v3.6.1 genome assembly (http://www.melonomics.net) were used to identify the variants (SNPs and INDELs). Fast-GBS uses the maximal exact matches algorithm implemented in BWA for alignment of the reads, and relies on the Platypus software [35] for variant calling. The parameters applied were the following: minimum number of reads per locus (default = 2); mapping quality score of reads to call a variant (MQ ≥ 20); minimum base quality (20); multiple nucleotide polymorphisms distance (minFlank = 5), and maximum missing data allowed (default ≤80%). As a preliminary check, the row data were cross-checked with a set of variants identified after the whole genome re-sequencing of the two parental lines PS and Ved [36], with the overlapping variants retained (pre-filtered variants) and used in the downstream analyses. Vcftools [37] and in-house scripts were subsequently applied to retain only bi-allelic variants with a Minor Allele Count greater than 1, with at least one homozygous variant for marker, with a global quality greater than 100 and with missing values (MV) ≤ 60%. In addition, sparse heterozygous variants with a genotype depth lower than 5 were converted to missing values.

Once variants were obtained and filtered, a chromosome-by-chromosome manual inspection was performed and variants showing highly distorted segregation and discrepancies with the genotyping of the parental lines were discarded. This final set of variants was used to define the bins. A bin was established as a group of variants with the same genotype for each RIL, meaning a region without any recombination breakpoint in any individual of the population. A single variant with the lowest number of missing data was selected to represent each bin. If missing data were present in this variant, they were imputed manually, recovering the genotyping information from the other variants in the same bin. Finally, only bins with less than 30% of missing data were considered valid to generate the genetic map. Bins with a discrepancy between genetic and physical maps but showing a proper segregation and reliable quality were included in the genetic map construction.

The genetic linkage map was constructed using the online software tool MSTmap [38]. Linkage groups (LG) were formed at minimum LOD = 10 and we allowed the software to detect genotyping errors. Mapping size threshold was set to 2 and the distance threshold to 15 cM. The genetic distances were estimated using the Kosambi mapping function [39]. The graphical representation of the genetic map was obtained using MapChart version 2.2 [40].

### QTL mapping

For quantitative traits, we were aware that environmental effects can have a considerable influence on the phenotype. A Principal Component Analysis (PCA) was used to test if the quantitative data of the blocks from the same year could be merged, creating only one dataset per year (Fig. 1a). We could not identify a pattern that distinguished the blocks according to the year, in fact T1 and T5 were very similar even though they were from different years. Considering these results, we decided to analyze the data in two different ways: one including the mean for each line after merging data from the five blocks, and the other including the individual data for each block.

The QTL mapping was performed with MapQTL6 [41] considering each block (T1-T5) and the mean across the blocks. We used the interval mapping procedure for all traits, and the Kruskal-Wallis (KW) test for the monogenic and oligogenic traits. QTLs in the mapping experiment with the mean values for LOD > 2.5 were considered significant. To evaluate their significance in the individual blocks we checked if they were located in the same position and with the same positive or negative additive effect.

The identified QTL, were coded with the terminology system described in [21], where the first letters represent the trait abbreviation, followed by a “Q” for QTL, a letter for the mapping experiment (“U”, in this case), a digit designating the linkage group (LG) to which the QTL maps, a dot and a final number to differentiate QTL present in the same LG.

### Statistical analyses

The statistical analyses and graphical representations were performed using the software R v3.2.3 [42] with the RStudio v1.0.143 interface [43].

The normality of distributions was tested using the Shapiro-Wilk test, assuming that it was significantly different from a normal distribution when *p*-value< 0.05.

The R package “factoextra” was used for PCA, with data from a random subset of 22 RILs which had no missing values in any block and a representative subset of seven quantitative variables (SSC, FW, FL, FD, FS, SN and SW). We removed the line effect to observe only the effect of the block (environment) before the PCA, using the “removeBatchEffect” function from the R package “limma”.

To obtain the correlation matrix among traits we calculated the Pearson coefficient with the R package “Hmisc” with “corrplot”.

## Additional files

Additional file 1: **Figure S1.** Distribution of the quantitative traits evaluated in the parental lines PS, Ved and Hyb. Each dot corresponds to an observation in any of the five blocks T1-T5. The mean for each line is shown with a horizontal line. (TIF 783 kb)

Additional file 2: **Figure S2.** Distribution of the quantitative traits evaluated in the RIL population for each block (T1-T5). Black stars indicate a significant deviation from normality. (TIF 2161 kb)

Additional file 3: **Table S1.** Markers obtained with GBS. (**A**) All markers; (**B**) Selected markers; (**C**) All bins; (**D**) Selected bins. (XLSX 5125 kb)

Additional file 4: **Figure S3.** Examples of complicated cases to phenotype. a. Mottled rind (MOT) is partially masked in PS due to the dark green rind, but darker spots can be observed in comparison to RIL 196A. b. Yellowing of mature rind (YELL), present in RIL 190 and appreciable as a different tone in green rind. (TIF 9290 kb)

Additional file 5: **Table S2.** Summary of QTLs described in other studies that map in similar intervals to those detected here. (PDF 80 kb)

Additional file 6: **Figure S4.** LOD scores for SSC in the QTL mapping experiment using the mean values for a QTL region in LG VIII. The dots represent the LOD peak for each QTL (*SSCQU8.1*, *SSCQU8.2* and *SSCQU8.3* from left to right) and the bars the LOD peak ±1.5. (TIF 159 kb)

Additional file 7: **Figure S5.** RILs 172 (a) and 140 (b) from 2016, showing transgressive segregation in fruit size in comparison with the parental lines PS (c) and Ved (d). The white square represents 1cm^2^. (TIF 9704 kb)

Additional file 8: **Table S3.** (a) Candidate genes for fruit morphology in the melon genome annotation v4.0. (b). Candidate genes for fruit morphology contained in the QTL intervals. (XLSX 19 kb)

Additional file 9: Phenotyping data values for each trait. (TXT 5 kb)

Additional file 10: Genotyping data. (TXT 157 kb)

Additional file 11: Linkage group positions for each marker. (TXT 18 kb)

## Abbreviations

CAR: Total carotenoids
DAP: Days after pollination
ECOL: External color of immature fruit
FD: Fruit diameter
FL: Fruit length
FP: Fruit perimeter
FS: Shape index
FW: Fruit weight
GBS: Genotyping-by-sequencing
IL: Introgression line
KW: Kluskal-Wallis
LG: Linkage group
MOT: Mottled rind
MQ: Mapping quality
MV: Missing value
NGS: Next-generation sequencing
PCA: Principal component analysis
PS: Piel de sapo
QTL: Quantitative trait locus
RIL: Recombinant inbred line
SN: Seed number
SSC: Soluble solid content
SW: Seed weight
VED: Védrantais
YELL: Yellowing of mature rind
