# Supplementary figures and images for: QTL mapping of melon fruit quality traits using a high-density GBS-based genetic map
Source: BMC Plant Biol. 2018 Dec 4;18:324. doi: 10.1186/s12870-018-1537-5 (PMC6278158; doi:10.1186/s12870-018-1537-5)

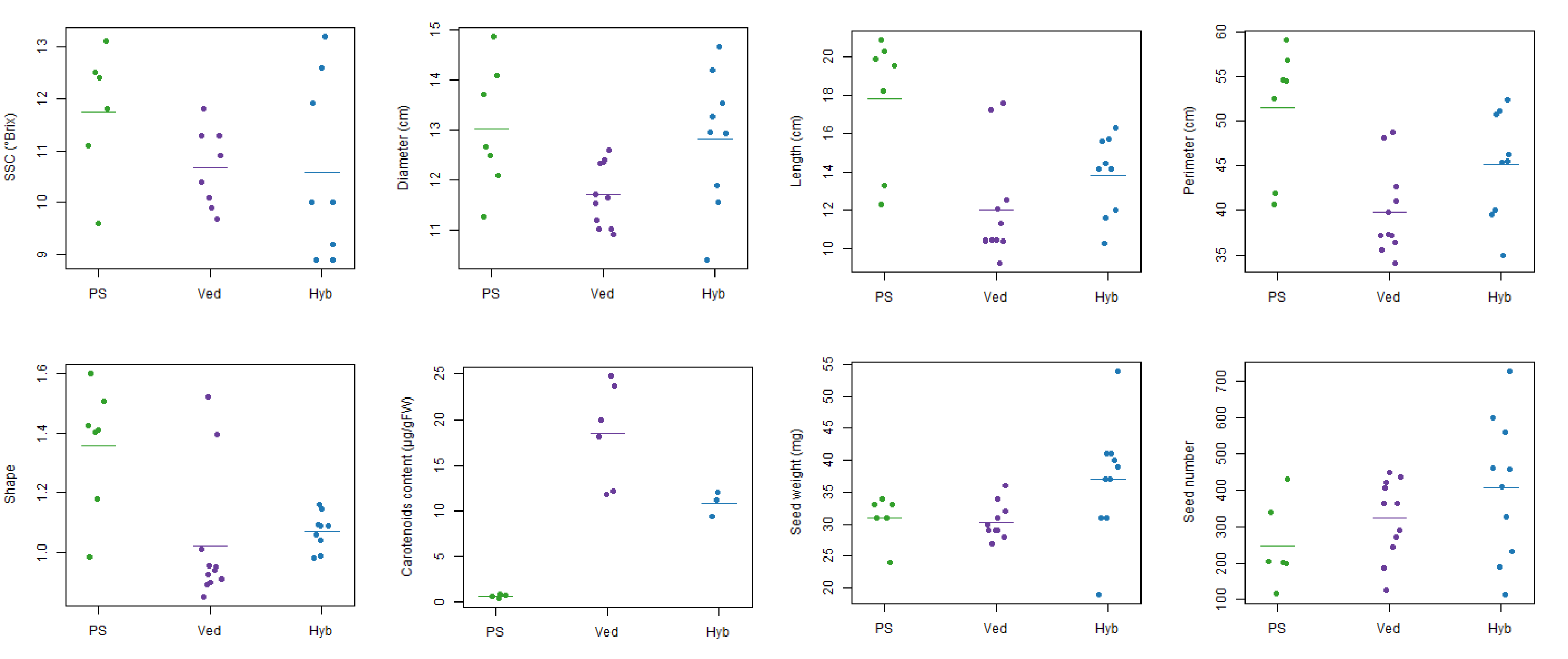

Supplement: Supplementary file 1 — Figure S1. Distribution of the quantitative traits evaluated in the parental lines PS, Ved and Hyb. Each dot corresponds to an observation in any of the five blocks T1-T5. The mean for each line is shown with a horizontal line. (TIF 783 kb) [file 12870_2018_1537_MOESM1_ESM.tif]

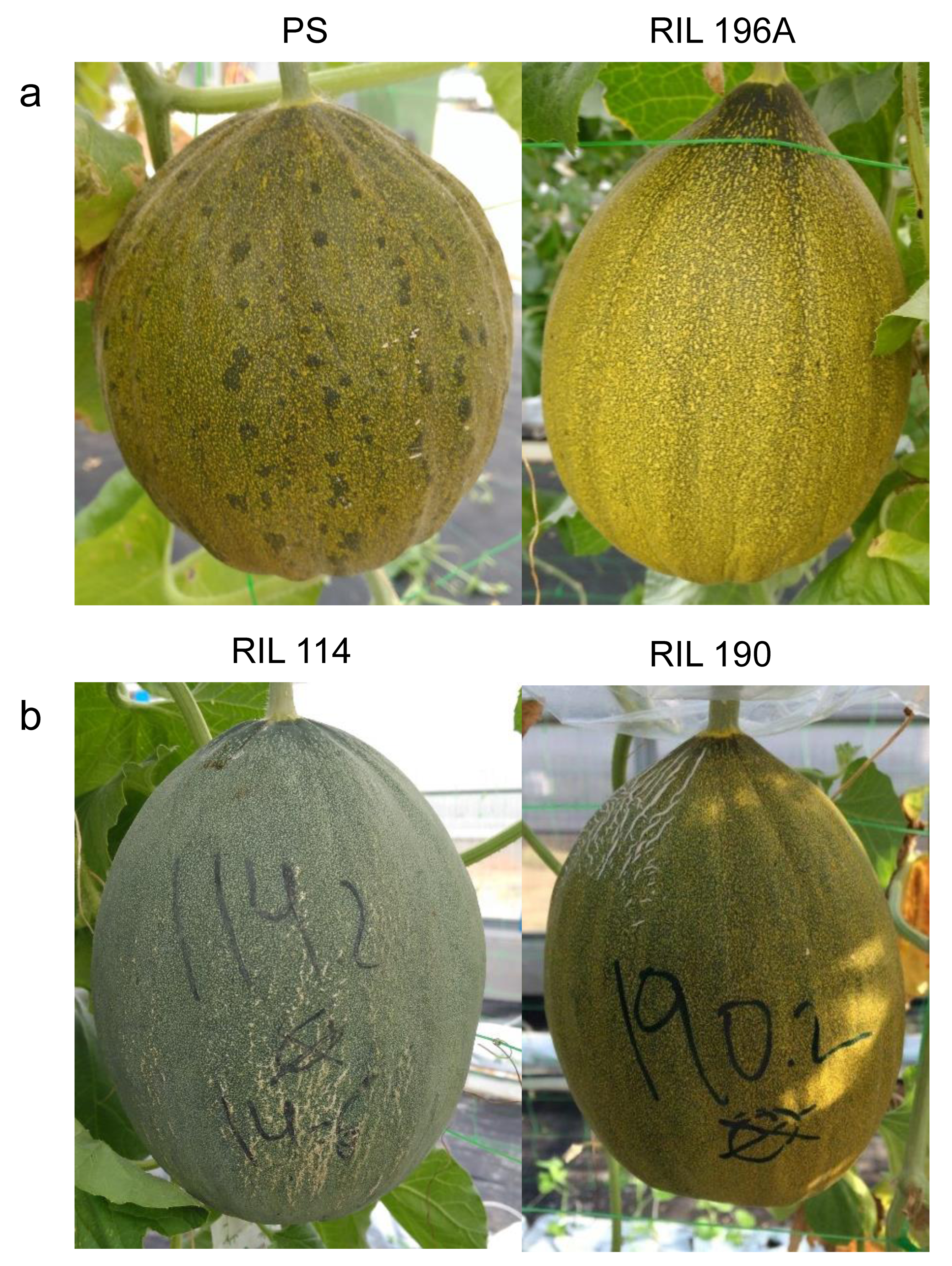

Supplement: Supplementary file 4 — Figure S3. Examples of complicated cases to phenotype. a. Mottled rind (MOT) is partially masked in PS due to the dark green rind, but darker spots can be observed in comparison to RIL 196A. b. Yellowing of mature rind (YELL), present in RIL 190 and appreciable as a different tone in green rind. (TIF 9290 kb) [file 12870_2018_1537_MOESM4_ESM.tif]

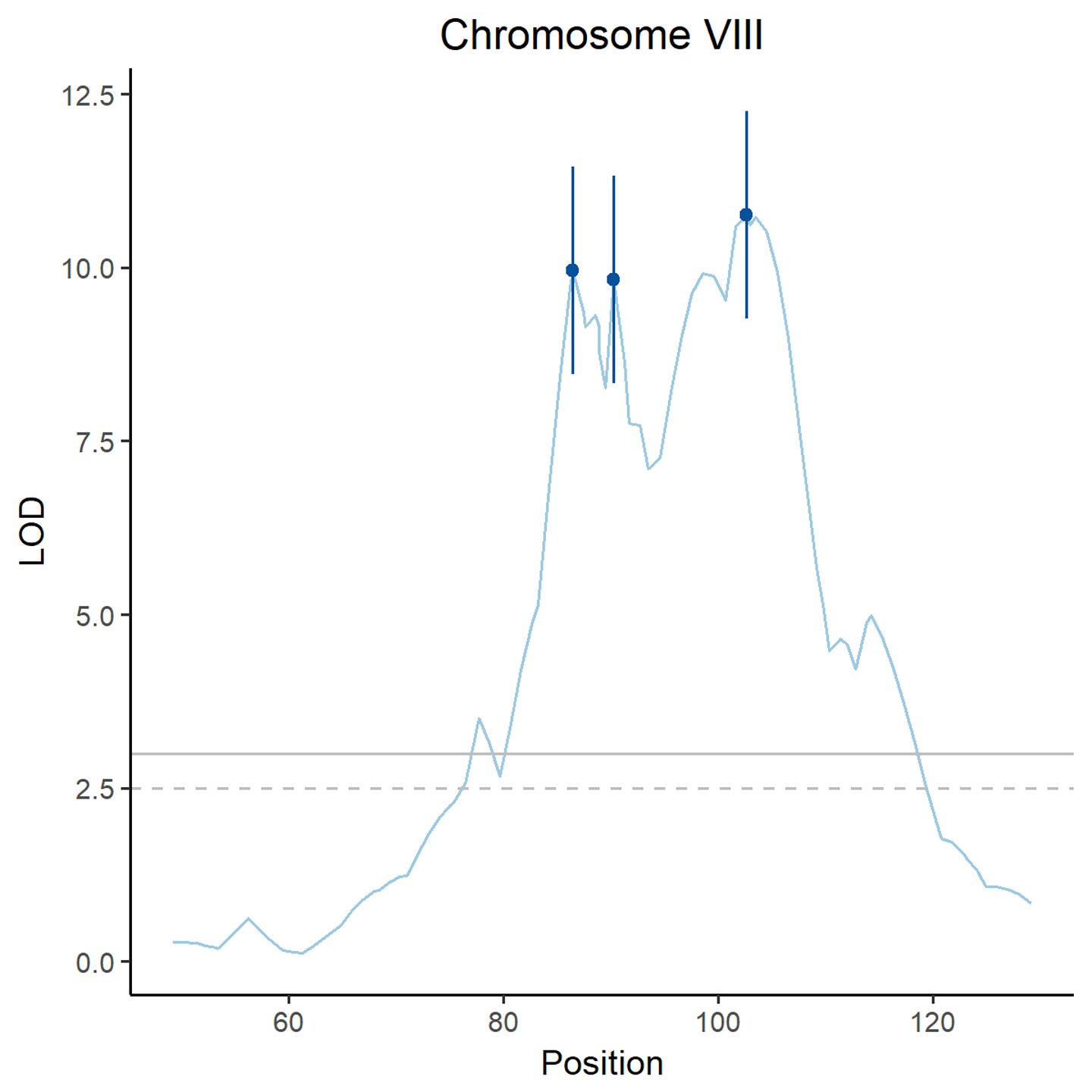

Supplement: Supplementary file 6 — Figure S4. LOD scores for SSC in the QTL mapping experiment using the mean values for a QTL region in LG VIII. The dots represent the LOD peak for each QTL (SSCQU8.1, SSCQU8.2 and SSCQU8.3 from left to right) and the bars the LOD peak ±1.5. (TIF 159 kb) [file 12870_2018_1537_MOESM6_ESM.tif]

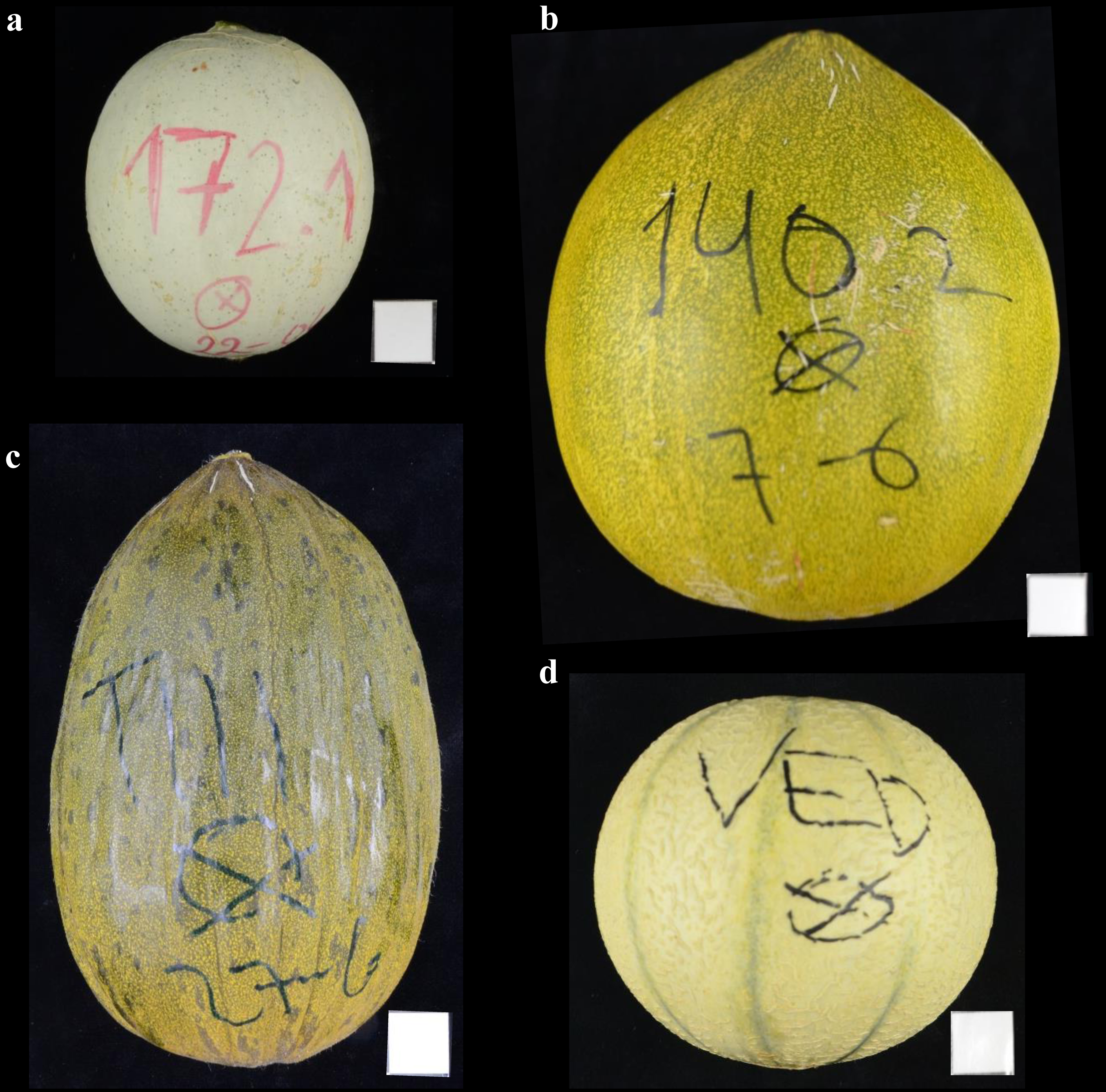

Supplement: Supplementary file 7 — Figure S5. RILs 172 (a) and 140 (b) from 2016, showing transgressive segregation in fruit size in comparison with the parental lines PS (c) and Ved (d). The white square represents 1cm2. (TIF 9704 kb) [file 12870_2018_1537_MOESM7_ESM.tif]
