# Supplementary material for: QTL mapping of melon fruit quality traits using a high-density GBS-based genetic map
Source: BMC Plant Biol. 2018 Dec 4;18:324. doi: 10.1186/s12870-018-1537-5 (PMC6278158; doi:10.1186/s12870-018-1537-5)
Supplement: Supplementary file 5 — Table S2. Summary of QTLs described in other studies that map in similar intervals to those detected here. (PDF 80 kb) [file 12870_2018_1537_MOESM5_ESM.pdf]

Trait	QTL/gene ID	Plant material	Chr	Physical position[1] (bp)	Linked marker	Principal reference[2]	Other references
SSC	*SSC8.5*	"Top Mark" x USDA-846-1	8	-	OPAY1-831	[78]	[59,66]
	SSC9.7	"Top Mark" x USDA-846-1	9	24,516,921	CMATN22	[78]	[66]
	SC9-3	PS x SC	9	-	CMCT1b	[66]	
	SSC10.8	"Top Mark" x USDA-846-1	10	3,803,151	CMGA172	[78]	
FW	*FWQT8.1*	PS x TRI	8	4,310,341	CI_33-B09	[68]	[5,66]
	SC5-2	PS x SC	5	20,855,850	CMGAN3	[66]	[59]
FD	*FD.2*	MAK x VED	2	8,178,416	AI_14-H05	[6]	[5,78,79]
FS	*FSQT6.1*	PS x TRI	6	15,162,313	AI_19-F11	[68]	[5,11,59,66,78]
	FS.11	MAK x VED	11	29,558,732	PSI_41-B07	[6]	[11,59,78,79]
	FSH2.1	PI 414 x "Dulce"	2	900,871	CMAGN39	[22]	[5,11,78,79]
FL	*FLQT6.1*	PS x TRI	6	36,413,356	CMPSNP1021	[68]	[4,6,66]
	FL.11	MAK x VED	11	31,483,477	CMPSNP389	[6]	[21,66]
	FL5.1	VED x PI 414	5		M35_14	[79]	
YELL	*CmKFB*	NA x TVT	10	3,475,283	-	[46]	
ECOL	*ECOLQC3.5*	PS x SC	3	3,914,573	CSWCT10	[59]	[21]
	ECOLQC7.2	PS x SC	7	23,437,923	CMTCN30	[59]	[21]
CAR	*CmOr*	"Dulce" x "Tam Dew"	9	21,683,406	-	[47]	

[1]When possible, the physical position in v3.6.1 of the melon genome is presented, based on the principal reference

[2]The most recent reference is indicated when the QTL was described multiple times
